# Comparative Risks of Pneumonitis Amongst Immune Checkpoint Inhibitors in Patients with Lung Cancer: A Network Meta-Analysis of Randomized Clinical Trials

**DOI:** 10.3390/ph19020219

**Published:** 2026-01-27

**Authors:** Ruba Abdel Razzaq Ebzee, Ibrahim Yusuf Abubeker, Ahmed Aboughalia, Mohammed I. Danjuma

**Affiliations:** 1 Division of Internal Medicine, Hamad Medical Corporation, Doha P.O. Box 3050, Qatar; rubaabdulrazaq@yahoo.com (R.A.R.E.); aaboughalia@hamad.qa (A.A.); 2 Mayo Clinic Rochester, Rochester, MN 55902, USA; abubeker.ibrahimyusuf@mayo.edu; 3 Internal Medicine Residency Program, Hamad Medical Corporation, Doha P.O. Box 3050, Qatar; 4 College of Medicine, Qatar University, Doha P.O. Box 2713, Qatar; 5 Weill Cornell College of Medicine New York, Doha P.O. Box 24144, Qatar; 6 NHS Grampian, Dr. Grays Hospital, Elgin IV30 1SN, UK

**Keywords:** pembrolizumab, nivolumab, immune checkpoint inhibitors, network, meta-analysis

## Abstract

**Background/Objectives:** Despite immune checkpoint inhibitors (ICPIs)’s transformation of lung cancer treatment, pneumonitis remains a potentially serious immune-related adverse event. However, reliable data on the comparative risks of individual ICPIs remain unknown. We conducted this network meta-analysis (NMA) to, therefore, quantify and compare the exact pooled burden of pneumonitis risk across multiple ICPI analogs. **Methods:** We searched the following databases, PubMed, Embase, Scopus MEDLINE and Cochrane Database of Systematic Reviews, as well as gray literature on Google Scholar for eligible studies reporting on the prevalence of pneumonitis following immune check point inhibitor exposures. Pairwise and network meta-analyses were performed to estimate pooled odds ratios (ORs) for pneumonitis, using placebo as the common comparator. Sensitivity analyses assessed the impact of study quality and combination therapies. **Results:** A total of 29 studies enrolling 15,271 patients with non-small cell lung cancer (NSCLC) or small-cell lung cancer (SCLC) satisfied the inclusion criteria and are included in the meta-analysis. Pembrolizumab was associated with a significantly increased risk of pneumonitis compared to placebo (odds ratio [OR] = 2.67, 95% confidence interval [CI]: 1.70–4.17), with similar elevated risk observed for sugemalimab (odds ratio [OR] = 2.45, 95% confidence interval [CI]: 1.52–3.95). Nivolumab was associated with increased odds of pneumonitis, although with unstable point estimate (odds Ratio [OR] = 2.69, 95% confidence interval [CI]: 0.64–11.35). Statistical heterogeneity was low (*H* statistics = 1.34). Atezolizumab and ipilimumab demonstrated modest or uncertain risk. Heterogeneity was low (*I*^2^ = 12%), and results were robust to sensitivity analyses. Higher pneumonitis rates were observed in combination regimens. **Conclusions:** Our analysis demonstrates that pneumonitis risk varies among ICPIs, with pembrolizumab and sugemalimab showing the highest odds. Although the absolute incidence is low, the potential severity of pneumonitis warrants vigilant monitoring. These results should guide clinicians in risk stratification and treatment planning, and they should support the development of standardized reporting criteria and further comparative research.

## 1. Introduction

Immune checkpoint inhibitors (ICPIs) have significantly improved clinical outcomes in patients with non-small cell lung cancer (NSCLC) and small-cell lung cancer [1,2,3,4]. They inhibit immunologic checkpoints, thereby restoring T-cell activation and antitumor immunity in patients with cancer [1]. Pembrolizumab and nivolumab are monoclonal antibodies that block the interaction between programmed cell death 1 and its ligands, PD-L1 and PD-L2 [1], whereas atezolizumab, sugemalimab, and durvalumab are monoclonal antibodies that bind programmed death ligand 1, blocking its engagement with PD-1. Ipilimumab is a monoclonal antibody that binds cytotoxic T-lymphocyte antigen-4, blocking its interaction with CD80 and CD86 [1]. Despite this significant improvement in clinical outcomes with the advent of these agents, immune-related adverse events (irAEs) such as pneumonitis continue to pose serious clinical challenges. Pneumonitis, while rare, can lead to significant morbidity and mortality, particularly in patients with pre-existing pulmonary comorbidities or those receiving combination therapies [1]. The aggregate of current evidence suggests varying pneumonitis risks across different ICPIs, yet direct comparisons remain limited. Previous pairwise meta-analyses and pharmacovigilance reports have indicated potential differences in toxicity profiles between PD-1 and PD-L1 inhibitors [2]. However, these analyses often lack the scope and statistical rigor of network-based approaches, especially when those approaches are based not only on the broad adverse effects burden of ICPIs but rather on specific morbidities such as pneumonitis [5]. Emerging nanotechnology-based therapies, including nanoparticle-mediated drug delivery systems, are being explored to improve the therapeutic index of lung cancer treatments by enhancing tumor targeting and reducing systemic toxicity [6]. These innovations underscore a broader need for treatment platforms that maintain anticancer efficacy without the substantial immune-mediated toxicities—such as pneumonitis—currently observed with ICPIs.

Reported pneumonitis incidence varies widely across trials and observational datasets due to differences in diagnostic criteria, radiologic confirmation, grading approaches, and attribution practices [7]. Long-term experience with nivolumab, for example, demonstrates delayed presentation of pneumonitis but also highlights inconsistencies in how it is monitored and reported across studies [4]. More recent first-line immunotherapy trials, including CheckMate-227, illustrate substantial heterogeneity in the capture of pulmonary toxicities and variation in the thresholds used to classify and confirm pneumonitis-related events [8]. Retrospective studies add further limitations, including small cohort sizes and incomplete imaging review, as well as diagnostic uncertainty when distinguishing pneumonitis from infection, tumor progression, or radiation-induced lung injury [9,10]. Mortality estimates for severe pneumonitis remain imprecise; findings, such as the high mortality reported by Tone et al., are derived from single-center experiences subject to referral and selection biases [11]. Evidence guiding rechallenge is likewise constrained, as safety data predominantly reflect highly selected patients fit for retreatment after grade ≥ 2 irAEs [12]. Broader irAE registries also exhibit reporting heterogeneity, with variable inclusion of low-grade or subclinical cases and inconsistent clinician-reported grading [2]. Related toxicities such as ICPI-associated myocarditis further demonstrate the spectrum of immune-mediated injury, yet mechanistic insight and biomarker-based risk stratification remain limited [13]. These collective limitations underscore the need for more standardized, prospective, and mechanistically grounded characterization of ICPI-related pneumonitis to enhance early detection and clinical management.

Immune checkpoint inhibitor-associated pneumonitis (ICI-pneumonitis) represents one of the most clinically significant immune-related adverse events of immunotherapy [9]. Although relatively uncommon, occurring in approximately 2–5% of treated patients, it carries disproportionate morbidity and mortality [7,10]. Severe (grade ≥ 3) cases occur in about 0.8%, frequently necessitating immunotherapy discontinuation, hospitalization, and high-dose corticosteroid therapy [11,12,13]. Fatal outcomes are reported in up to 30% of severe cases [14,15]. The burden extends beyond patient outcomes, as ICI-pneumonitis often mimics infection, radiation injury [16], or tumor progression, creating diagnostic uncertainty and delaying cancer therapy. Additionally, real-world data show higher incidence rates compared with clinical trials [17], likely to reflect broader patient comorbidities and combined ICI regimens. Given the expanding use of ICIs across malignancies, awareness, early detection, and standardized management are crucial to mitigate this growing clinical and healthcare burden. Despite this enormous burden and ever-present uncertainty regarding the type of patients who are likely to develop immune-related ICPI complications, outcomes from primary studies have been conflicting. Even subsequent pooled analyses, such as the meta-analytical synthesis by Su et al. [18], still left a lot of unresolved concerns regarding the exact numerical comparative pneumonitis burden of various ICPI analogs in patients in these cohorts of patients. Although other factors, such as performance in RCT as well as affordability, will rank higher on the factors that determine the choice of individual ICPIs, having additional information on the comparative risks of IMAE (such as pneumonitis) will additionally support this attempt at risk stratification. Furthermore, since the publication of the last meta-analysis, additional studies have been published, further providing additional data points for the comparative determination of risk [4,5,8,12].

In this network meta-analysis, we have carried out comparative pooled risk assessment on pneumonitis through both direct and indirect comparison between various ICPIs (such as pembrolizumab). This is with the view to determining the exact pneumonitis burden of each and, in this way, guide therapeutic choices. This study addresses limitations of prior reviews by including a broader set of studies and applying sensitivity analyses to assess the robustness of our resulting point estimates (across study designs, treatment contexts, and patient subgroups).

## 2. Materials and Methods

The systematic review protocol was registered in the International PROSPERO database (International Prospective Register of Systematic Reviews; https://www.crd.york.ac.uk/PROSPERO/view/CRD420251079154 (assessed on 23 June 2025)) under the registration number CRD420251079154. Registration was completed prior to data collection and analysis. All methods and inclusion criteria were implemented as initially planned. This is systematic review and network meta-analysis was conducted in compliance with the PRISMA guidelines.

### 2.1. Search Strategy

A comprehensive literature search was undertaken to identify randomized controlled trials evaluating pneumonitis risk associated with immune checkpoint inhibitors (ICPIs) in patients with lung cancer. The search was conducted across multiple electronic databases, including PubMed, Embase, Scopus, MEDLINE, and the Cochrane Database of Systematic Reviews, from database inception to June 2025. In addition, gray literature sources, such as Google Scholar and trial registries (ClinicalTrials.gov and WHO ICTRP), were reviewed to ensure completeness and minimize publication bias. The search combined Medical Subject Headings (MeSH) and free-text terms relating to the interventions and outcome of interest. The final search syntax was structured as follows: (“immune checkpoint inhibitors” OR “PD-1” OR “PD-L1” OR “CTLA-4”) AND (“pneumonitis” OR “interstitial lung disease” OR “lung toxicity”) AND (“randomized controlled trial”) AND (“lung cancer” OR “SCLC” OR “NSCLC”). The reference lists of all included articles and relevant review papers were manually screened to identify additional eligible studies.

### 2.2. Eligibility Criteria

Eligible studies were randomized controlled trials that enrolled adult patients (≥18 years) with histologically confirmed non-small cell lung cancer (NSCLC) or small-cell lung cancer (SCLC) who received treatment with at least one immune checkpoint inhibitor targeting PD-1, PD-L1, or CTLA-4 pathways, either as monotherapy or in combination with other agents. Studies were required to report extractable data on the incidence or number of pneumonitis cases of any grade, as defined by Common Terminology Criteria for Adverse Events (CTCAE). Trials that compared ICPIs with placebo, standard chemotherapy, or other ICPIs were included. Exclusion criteria were preclinical or non-oncologic studies, trials enrolling pediatric populations, studies involving non-randomized or single-arm designs, and those that did not provide sufficient data to calculate pneumonitis event rates or effect estimates.

### 2.3. Study Selection and Data Extraction

All retrieved citations were imported into the Rayyan QCRI software (Rayyan QCRI; Qatar Computing Research Institute, Hamad Bin Khalifa University, Doha, Qatar) for reference management and duplicate removal. Two reviewers (RE and MID) independently screened titles and abstracts to identify potentially eligible studies. The full texts of all potentially relevant articles were then obtained and reviewed in detail. Disagreements between reviewers regarding inclusion or exclusion were resolved by consensus, and if unresolved, arbitration was undertaken by a third reviewer (AA).

A standardized data extraction form was developed and piloted on ten randomly selected studies to ensure uniformity and reliability. The extracted information included details on study design, publication year, country, phase, sample size, histologic subtype, intervention type, comparator arm, ICPI dosage and regimen, follow-up duration, and the number of pneumonitis cases (any grade and grade ≥ 3, where available). When essential data were missing or unclear, corresponding authors were contacted to obtain clarification.

### 2.4. Risk of Bias Assessment

The methodological quality of the included studies was evaluated using the Cochrane Risk of Bias 2.0 tool, and the results were visualized using the ROBVIS web application (developed by Evidence Synthesis Ireland and the Centre for Open Science, Galway, Ireland and Bristol, UK). This tool assesses the internal validity of randomized trials across six core domains: the randomization process, deviations from intended interventions, completeness of outcome data, accuracy of outcome measurement, selective reporting, and other potential sources of bias. Each domain was rated as low, some concerns, or high risk of bias. Two reviewers (AA and MID) performed independent assessments, and discrepancies were resolved through discussion. Studies classified as having a high risk of bias were subjected to sensitivity analyses to determine their influence on pooled estimates.

### 2.5. Network Geometry and Treatment Nodes

A network plot was constructed to illustrate the geometry of treatment comparisons among the included trials. Each node represented a distinct intervention, and edges denoted direct head-to-head comparisons between treatments. The node size was weighted according to the number of participants who received that particular treatment, while the thickness of each connecting line reflected the number of trials contributing to that comparison. Placebo served as the reference node, forming the largest and most interconnected point within the network. The network comprised seven active ICPIs: pembrolizumab, nivolumab, durvalumab, atezolizumab, avelumab, sugemalimab, and ipilimumab. The geometry revealed a well-connected, star-shaped structure centered on placebo, ensuring the feasibility of indirect comparisons across agents. There were no disconnected components or closed loops that would threaten the transitivity assumption required for network meta-analysis.

### 2.6. Statistical Analysis

All analyses adhered to PRISMA guidelines for network meta-analyses. The primary outcome was the odds ratio (OR) for any-grade pneumonitis associated with ICPI therapy. Initially, pairwise meta-analyses were performed for comparisons between each ICPI and placebo using a random-effects DerSimonian–Laird model to account for between-study heterogeneity. Heterogeneity was quantified using the *I*^2^, *Q*, and *H* statistics, with an *I*^2^ value exceeding 50% indicating substantial heterogeneity.

Subsequently, a frequentist random-effects network meta-analysis (NMA) was undertaken to estimate pooled odds ratios and 95% confidence intervals using placebo as the common comparator. The relative ranking of each intervention was determined using P-scores, which estimate the probability that a given treatment is among the most effective or least harmful options. The assumption of transitivity was verified by ensuring comparability of clinical and methodological characteristics across trials.

Consistency between direct and indirect evidence was examined using the design-by-treatment interaction model and node-splitting approaches, with *p*-values > 0.05 indicating satisfactory global and local consistency, respectively. Usually, network forest plots are generated based on Bayesian methods that rank treatments by the surface under the cumulative ranking curve (usually called SUCRA). From a frequentist perspective (which was our methodology), treatment effects are thought of as fixed parameters and thus, strictly speaking, a concept like SUCRA does not apply. A frequentist alternative called the P-score was proposed, but SUCRA or P-scores have no major advantage compared to ranking treatments by their point estimates [19]. Rather, treatments are ranked by their effect sizes in the network forest plot, with a dot in the plot giving the central estimate, and the line representing the confidence interval. Sensitivity analyses were conducted to evaluate the robustness of results, excluding studies with a high risk of bias and those evaluating combination regimens, thereby isolating the effects of monotherapy. Additional subgroup analyses were stratified by cancer type (NSCLC versus SCLC), ICPI class (PD-1 versus PD-L1 versus CTLA-4 inhibitors), and trial phase. Publication bias was assessed through visual inspection of funnel plot symmetry and confirmed using Egger’s regression test. All analyses were carried out in MetaXL (version 5.3, EpiGear International, Queensland, Australia), R (netmeta package, R Core Team, Vienna, Austria; https://www.R-project.org, accessed on 5 December 2025), and Stata version 15 (metan and network commands; StataCorp LLC, College Station, TX, USA.

## 3. Results

### 3.1. Study Characteristics

**Figure 1:**
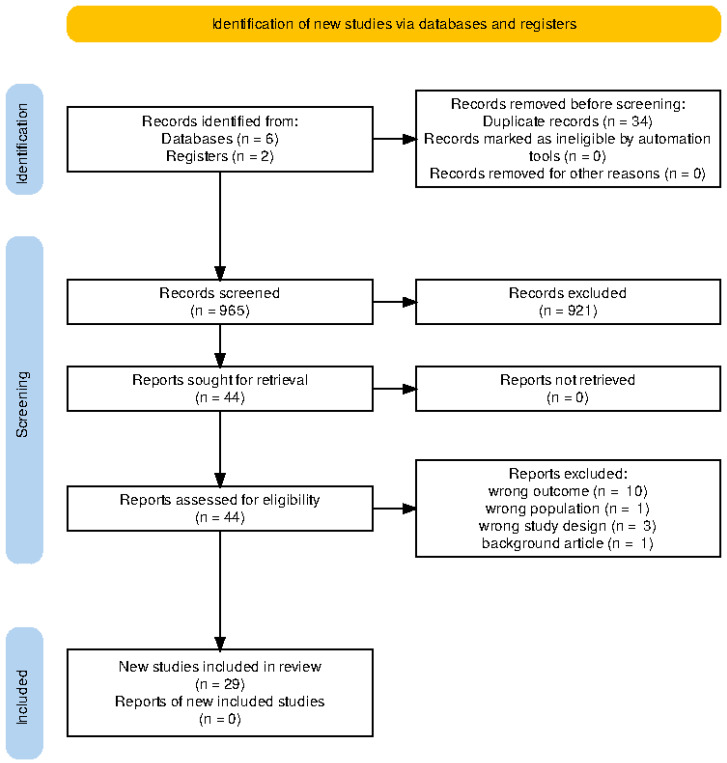
Total number of studies retrieved from the initial search of relevant registries and databases, as well as the final number of studies included in the review following exhaustive screening.

Table 1 shows the full socio-demographic characteristics of the included studies. Electronic search of relevant databases returned n = 965 titles and abstracts, from which n = 29 studies were eligible for inclusion into the systematic review and network meta-analysis (Figure 1). A total of 29 studies [20,21,22,23,24,25,26,27,28,29,30,31,32,33,34,35,36,37,38,39,40,41,42,43,44,45,46,47,48] were included in this network meta-analysis, encompassing patients primarily with non-small cell lung cancer (NSCLC), 82.76%, and a minority with small-cell lung cancer (SCLC), 17.2%. The majority of the studies were conducted in early-phase settings: Phase I (27.6%) [25,26,34,36,37,45], Phase II (62.1%) [20,21,22,23,24,30,31,32,33,35,39,42,43,46,47,48], and Phase III (10.3%) [28,38,39].

### 3.2. Prevalence Estimates Stratified by Type of ICPI

Regarding the types of immune checkpoint inhibitors (ICPIs) explored, pembrolizumab was the most frequently studied (40.7%), followed by nivolumab (22.2%), atezolizumab (14.8%), ipilimumab (11.1%), durvalumab (7.4%), and sugemalimab (3.7%), as shown in Table 1. Combination regimens, involving concurrent chemotherapy, radiotherapy, or dual ICPI therapy, reflect real-world treatment strategies and clinical trial complexity. The Common Terminology Criteria for Adverse Events (CTCAE) was used for adverse event grading in nearly all studies, although a few used modified criteria or did not specify the grading approach.

### 3.3. Pairwise Meta-Analysis (Pembrolizumab vs. Placebo)

**Figure 2:**
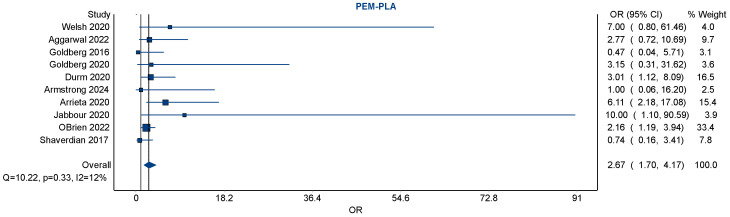
A forest plot of pairwise meta-analyses of studies exploring the risk of pneumonitis amongst NSCLC patients exposed to pembrolizumab vs. placebo (usual standard of care). There was negligible heterogeneity amongst the ten included studies (with an *I*^2^ of 12%). Pembrolizumab was associated with a significantly increased risk of pneumonitis, with a pooled odds ratio of 2.67 [95% CI 1.70–4.17] [20,23,24,25,34,35,41,42,43,47].

**Scheme A2:**
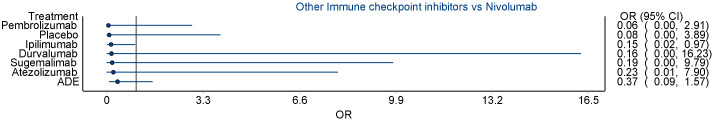
Figure showing comparative estimate between nivolumab and other ICPIs.

To estimate the risk of pneumonitis with pembrolizumab relative to placebo, a pairwise meta-analysis was conducted using 10 studies directly comparing these interventions. The pooled odds ratio was 2.67 (95% confidence interval [CI]: 1.70 to 4.17), indicating that pembrolizumab was associated with a statistically significant increase in the risk of pneumonitis (Figure 2). This analysis incorporated a broad range of study populations and treatment settings, including both monotherapy and combination regimens. Additional comparison between other ICPIs and placebo is shown in the Appendix A (Scheme A2).

The heterogeneity among studies was low, with an *I*^2^ statistic of 12%, a *Q*-statistic of 10.22 (*p* = 0.33), and an *H*-statistic of 1.06, suggesting good consistency and minimal between-study variance. The relatively narrow confidence interval, along with the low heterogeneity, supports the reliability of this finding across multiple trials.

### 3.4. Small Study Effect and Publication Bias

Visual inspection of the forest plot showed that although individual study effect estimates varied, they consistently favored an increased risk with pembrolizumab over placebo. A notable contributor to the weight of the analysis was the large RCT (O’Brien 2022) [41], which alone accounted for over 33% of the pooled estimate, demonstrating the influence of large trials in driving overall findings. Smaller studies contributed less weight but did not significantly deviate from the overall trend.

### 3.5. Network Meta-Analysis (NMA)

**Figure 3:**
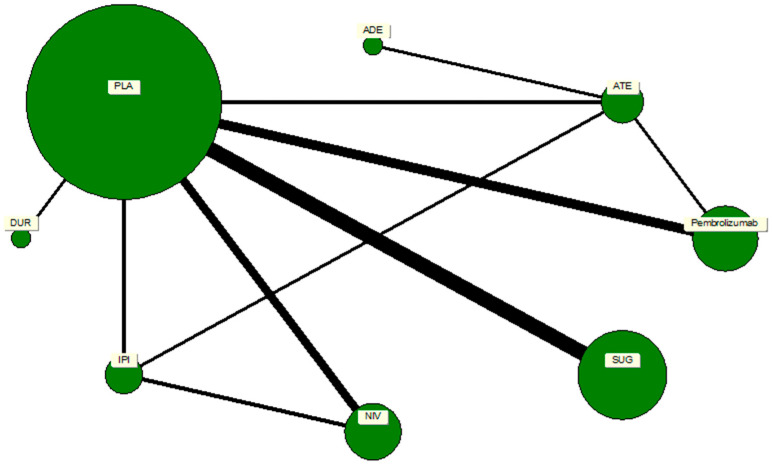
A network map of direct and indirect comparisons of the immune checkpoint inhibitor analogs. The size of the nodes depicts the number of studies associated with a particular drug or treatment regimen, whereas the thickness of the connecting strands indicates the number of studies associated between the two drugs.

A comprehensive network meta-analysis was conducted to compare the pneumonitis risk across all ICPIs, incorporating both direct and indirect comparisons. The network plot revealed a highly connected network centered on placebo, which was compared directly or indirectly with nearly every other agent. This strong connectivity bolstered the statistical robustness of the indirect comparisons, as in Figure 3.

Direct effect estimates from the NMA highlighted pembrolizumab, nivolumab, and sugemalimab as being associated with higher odds of pneumonitis relative to placebo. Specifically, pembrolizumab had an odds ratio of 2.67 (95% confidence interval [CI]: 1.70 to 4.17), nivolumab had an odds ratio of 2.69 (95% confidence interval [CI]: 0.64 to 11.35), and sugemalimab had an odds ratio of 2.45 (95% confidence interval [CI]: 1.52 to 3.95). These findings suggest that these agents pose a notably elevated risk for pneumonitis. However, confidence intervals for nivolumab were wider due to fewer direct comparisons, indicating less certainty in the point estimate, as in Table 2.

Other agents showed more modest or uncertain effects. Ipilimumab demonstrated an odds ratio of 1.93 (95% confidence interval [CI]: 0.06 to 59.87), while atezolizumab showed an odds ratio of 1.33 (95% confidence interval [CI]: 0.38 to 4.62). These results suggest potential but non-significant increases in risk, complicated by wide confidence intervals reflecting sparse data or high variance (all of which suggest instability of the point estimate). The indirect estimates comparing each ICPI to pembrolizumab revealed that sugemalimab had a comparable risk (odds ratio [OR] = 0.92, 95% confidence interval [CI]: 0.48 to 1.77), while atezolizumab was associated with a potentially lower risk (odds ratio [OR] = 0.50, 95% confidence interval [CI]: 0.13 to 1.87). Other agents, such as ipilimumab and durvalumab, demonstrated even lower odds compared to pembrolizumab, although these estimates were accompanied by extremely wide confidence intervals, limiting interpretability. Despite this, the consistency in the directionality of effect estimates suggests a potential hierarchy of pneumonitis risk, with pembrolizumab and sugemalimab at the upper end, as in Table 2.

### 3.6. Sensitivity Analyses

Sensitivity analyses were performed to assess the robustness of the primary findings. Exclusion of studies with a high risk of bias had minimal impact on the pooled estimates, with the odds ratio for pembrolizumab vs. placebo decreasing slightly but remaining statistically significant, suggesting that the observed association was not driven by study quality alone. Additional sensitivity analyses were conducted to account for the impact of combination therapies. When studies employing concurrent chemo-radiotherapy or dual ICPI regimens were excluded, the pooled odds ratio for pembrolizumab decreased modestly from 2.67 to approximately 2.1. This attenuation suggests that combination regimens may contribute modestly to pneumonitis risk, but that pembrolizumab independently confers an elevated risk.

Subgroup analyses by cancer type (NSCLC vs. SCLC) and by trial phase (Phase III vs. earlier) revealed broadly similar patterns of risk, though statistical power was limited due to small numbers in some strata. Overall, the direction and magnitude of effect remained consistent across sensitivity analyses, lending confidence to the main findings.

### 3.7. Heterogeneity and Publication Bias

**Figure 4:**
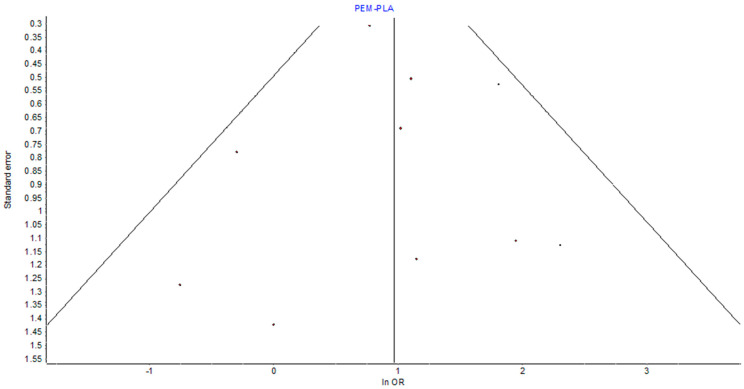
Funnel plot showing the effect of bias due to small studies. It shows the symmetry of the distribution of studies around the unity point (OR 1). This confirms the lack of significant small study effects on the overall point estimates.

The assessment of heterogeneity in the pairwise meta-analysis revealed low between-study variance, supported by an *I*^2^ of 12%, *Q* = 10.22 (*p* = 0.33), and *H* = 1.06. These values indicate that variation in effect sizes was likely due to chance rather than true heterogeneity. Visual inspection of the funnel plot for pembrolizumab vs. placebo showed general symmetry, providing no strong evidence of small study effects or publication bias, as in Figure 4.

### 3.8. Pneumonitis Outcomes and Adverse Event Reporting

Across studies, the incidence of grade ≥ 3 pneumonitis ranged from 0% to as high as 7% in the pembrolizumab arms, confirming that pneumonitis, though infrequent, can be clinically significant and occasionally life-threatening. All but four studies [30,37,42,44] used CTCAE criteria for grading adverse events, promoting consistency in reporting across the dataset. Notably, combination regimens tended to report higher rates of pneumonitis than monotherapy studies, underscoring the importance of evaluating treatment context. These findings are relevant for clinical decision-making in tailoring treatment based on patient risk profiles and expected toxicity burden.

### 3.9. Risk of Bias Assessment Outcomes

**Figure 5:**
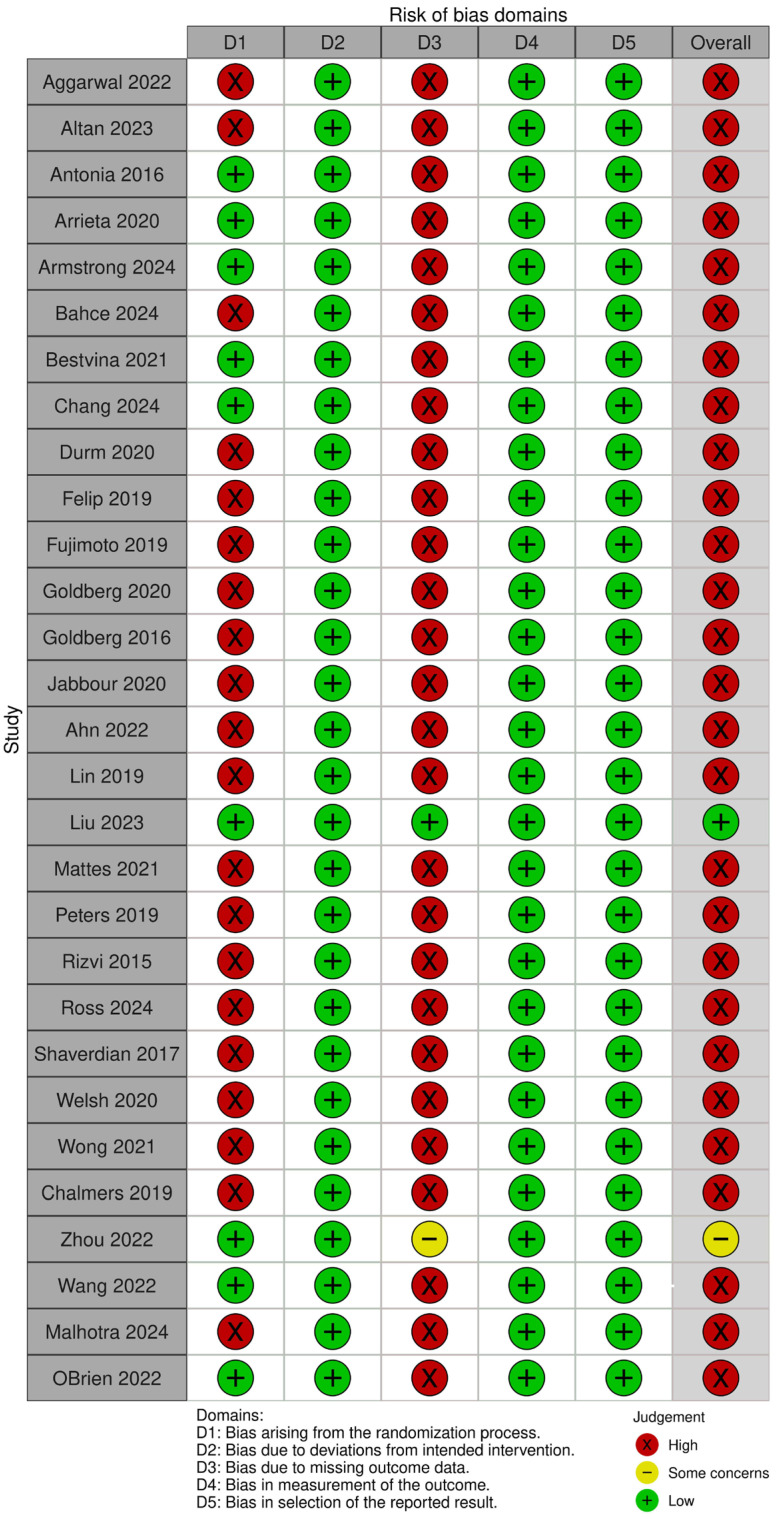
A pictorial representation of the risk of bias as a marker of methodological quality of the included studies across 5 domains. The green, red, and yellow color codes of the overall outcomes denote low, high, and moderate risks of bias, respectively [20,21,22,23,24,25,26,27,28,29,30,31,32,33,34,35,36,37,38,39,40,41,42,43,44,45,46,47,48].

Assessment of study quality revealed that 81.5% of studies were considered at high risk of bias, primarily due to open-label design, small sample sizes, or limited pneumonitis reporting. Only one large-scale RCT (Liu et al., 2023 [28]) was deemed to have a low risk of bias, while two other studies were rated as having some concerns, as shown in Figure 5.

## 4. Discussion

In this network meta-analysis of RCTs, we found increased pneumonitis risk associated with pembrolizumab compared to other ICPTs. This network meta-analysis provides a nuanced evaluation of pneumonitis risk across different immune checkpoint inhibitors (ICPIs) in lung cancer treatment, highlighting pembrolizumab and sugemalimab as having the highest relative risks. These findings build upon prior meta-analyses while offering new insights enabled by network-level comparisons and robust sensitivity testing. The increased pneumonitis risk associated with pembrolizumab (odds ratio [OR] = 2.67, 95% confidence interval [CI]: 1.70–4.17) aligns with several previous meta-analyses and pharmacovigilance studies. For example, a systematic review by Wang et al. [39]. found that PD-1 inhibitors, particularly pembrolizumab, were associated with a higher incidence of immune-related pneumonitis compared to PD-L1 inhibitors, potentially due to broader immune activation by PD-1 blockade. Our findings extend this knowledge by confirming the elevated risk through both direct and indirect comparisons across a larger and more heterogeneous patient population.

In contrast, the point estimate for nivolumab (odds ratio [OR] = 2.69) was also elevated but accompanied by a wide confidence interval (95% confidence interval [CI]: 0.64–11.35), reflecting imprecise estimation due to fewer direct comparisons. A similar trend was noted in the work of Ramalingham et al. [5], who observed that nivolumab carried a non-trivial pneumonitis risk, albeit slightly lower than pembrolizumab. Sugemalimab, a newer PD-L1 inhibitor, demonstrated a high odds ratio (odds ratio [OR] = 2.45, 95% confidence interval [CI]: 1.52–3.95), which may appear contradictory to the general perception that PD-L1 inhibitors carry a lower pneumonitis risk. However, its elevated risk in our analysis might be attributed to limited real-world safety data, smaller sample sizes, and the inclusion of high-risk populations in clinical trials. Additionally, sugemalimab trials have been conducted predominantly in Asian populations, and the existing literature suggests that pneumonitis rates may vary by ethnicity, with higher susceptibility reported among East Asian patients [5].

Atezolizumab and ipilimumab showed lower and less consistent associations with pneumonitis. Atezolizumab’s relatively favorable toxicity profile has been supported by prior pharmacovigilance reports and meta-analyses [33]. Ipilimumab, an anti-CTLA-4 antibody, has a distinct mechanism of action that may result in different immune-related adverse event patterns, with colitis and dermatitis often more prominent than pneumonitis [5,21].

Socio-demographic factors, though not consistently reported across studies, are important to consider in interpreting pneumonitis risk. Age is a recognized risk factor; older patients may have decreased pulmonary reserve. Males were overrepresented, and smoking status was inconsistently documented, both of which could influence observed toxicity rates.

These findings underscore the need for personalized risk–benefit assessments in ICPI therapy selection and for standardized adverse event reporting in future trials.

The pattern observed in our analysis, where pembrolizumab and sugemalimab demonstrated the highest odds of pneumonitis (OR 2.67 and OR 2.45, respectively), with nivolumab showing a similarly elevated but imprecise estimate, aligns with the underlying biological differences between PD-1 and PD-L1 blockade [49]. PD-1 inhibitors, such as pembrolizumab and nivolumab, exert broader immunologic disruption by simultaneously blocking PD-1 interactions with both PD-L1 and PD-L2 [49]. This dual inhibition is particularly relevant in the lung, where PD-L2 expression on airway epithelial and dendritic cells contributes to maintaining local immune tolerance and limiting excessive T-cell-mediated inflammation [50]. Interference with PD-1/PD-L2 signaling has been associated with enhanced Th1 and Th17 responses, increased interferon-γ activity, and greater susceptibility to alveolar injury—mechanistic pathways that plausibly explain the higher pneumonitis risk observed with PD-1 agents in our study [49,50]. In contrast, PD-L1 inhibitors, such as sugemalimab, block PD-L1 while preserving PD-1/PD-L2 signaling, theoretically conferring a more restricted immune activation profile [51,52]. The elevated pneumonitis risk we observed with sugemalimab suggests that PD-L1 blockade can still meaningfully perturb pulmonary immune homeostasis, particularly in patients with lung cancer, where background inflammation, tumor burden, or prior thoracic irradiation may amplify susceptibility. The modest or uncertain risk estimates seen with atezolizumab and ipilimumab are consistent with this graded biological model, and the higher pneumonitis rates in combination regimens reinforce the additive effect of intensified immune activation. Overall, our findings reflect and support the mechanistic hierarchy of pulmonary toxicity across ICPI classes, underscoring the need for vigilant monitoring, individualized risk assessment, and standardized pneumonitis-reporting frameworks.

In our network meta-analysis, each drug was individually evaluated not on the basis of its mechanism of action but as an individual analog of ICPI. Nonetheless, the point estimate of the burden of individual ICPIs as a representative class (for example, peembro as PD-1) that we found aligns with what is reported in the literature [8,11]. Monoclonal antibodies that bind programmed death ligand 1, blocking its engagement with PD-1, including atezolizumab, sugemalimab, and durvalumab, are associated with varying risks of pneumonitis. For example, atezolizumab had an odds ratio of 1.33 (95% confidence interval [CI]: 0.38 to 4.62), durvalumab had an odds ratio of 2.06 (95% [CI]: 0.17 to 23.88), and sugemalimab had an odds ratio [OR] of 2.45 (95% confidence interval [CI]: 1.52–3.95). Conversely, monoclonal antibody drugs that block the interaction between programmed cell death 1 and its ligands, PD-L1 and PD-L2 (including pembrolizumab and nivolumab), are associated with varying risks of pneumonitis [odds ratio [OR] of 2.67 (95% confidence interval [CI]: 1.70–4.17) and odds ratio [OR] of 2.69 (95% confidence interval [CI]: 0.64–11.35)], respectively. In general, PD-1 inhibitors were associated with a comparatively higher incidence of immune-related pneumonitis compared to PD-L1 inhibitors and the anti-CTLA-4 antibody, while the latter showed lower and less consistent associations with pneumonitis.

Several recent meta-analyses have sought to clarify the determinants of ICPI-related pneumonitis, yet each is limited by methodological constraints that temper the certainty of its conclusions [53,54,55]. Zhou et al. (2025) [55] synthesized risk factors for pneumonitis among lung cancer patients receiving immunotherapy; however, the studies contributing to their pooled estimates displayed considerable variability in pneumonitis definitions, imaging confirmation, and clinical attribution practices [55]. Many lacked multidisciplinary review or standardized grading, raising concerns regarding outcome misclassification and generating uncertainty around their reported point estimates. Li et al. (2024) [53] examined COPD as a potential modifier of pneumonitis risk, concluding that pre-existing lung disease confers higher susceptibility. However, the underlying evidence base was predominantly retrospective, with uneven reporting of pulmonary function metrics, emphysema burden, smoking exposure, and prior thoracic irradiation—all factors that may independently influence pneumonitis risk. Consequently, the observed association between COPD and CIP remains susceptible to residual confounding [53]. In contrast, our study addresses several of these recurring limitations by applying a rigorously defined pneumonitis outcome incorporating radiologic and clinical criteria, using standardized grading and adjudication procedures, and integrating more granular covariates reflective of real-world clinical complexity. This approach allows a more precise delineation of risk and provides a clearer framework for interpreting pneumonitis within contemporary immunotherapy practice.

The risk of bias assessment (Figure 5) showed that most included randomized trials had a high risk of bias in at least one domain, with several demonstrating concerns across D1 (randomization process) and D3 (missing outcome data). These domains are critical for outcome validity determination, especially in safety analyses where pneumonitis events are relatively infrequent and susceptible to detection and reporting variability. High risk in the randomization process raises the possibility of baseline imbalances or allocation bias, which could inflate or obscure treatment-related adverse events. Similarly, missing outcome data—often due to loss to follow-up or treatment discontinuation—may disproportionately affect pneumonitis ascertainment, thereby reducing confidence in the effect estimates. Despite these limitations, domains relating to the measurement of outcomes (D4) and selection of the reported result (D5) were generally adjudicated as low risk, reflecting consistent and protocol-driven reporting of pneumonitis across the trials

Our findings have several direct translational implications for both clinicians and regulatory agencies. Firstly, the observation that pembrolizumab and sugemalimab confer the highest pneumonitis risk, and with consistent directionality for nivolumab, provides an evidence-based foundation for potential risk-informed treatment selection. For oncologists, these results support the need for stricter pulmonary surveillance protocols, earlier radiologic assessment in symptomatic or high-risk patients, and heightened vigilance when initiating PD-1 or PD-L1 inhibitors in individuals with baseline lung morbidities or prior radiation therapy. Second, the low heterogeneity (*I*^2^ = 12%) and robustness of our sensitivity analyses strengthen the generalizability of these risk estimates across real-world practice, offering regulators a potentially more stable signal when evaluating post-marketing safety, updating product labels, or issuing risk-mitigation recommendations. Third, the consistently higher pneumonitis rates observed in combination regimens underscore the importance of adjusting trial monitoring requirements and mandating clearer attribution frameworks where radiotherapy, chemotherapy, and immunotherapy overlap. Collectively, the relevance of these findings lies in enabling more precise risk stratification, guiding surveillance intensity, and refining regulatory decision-making around the safe deployment of immune checkpoint inhibitors.

### Strengths and Limitations

Strengths of this study include the comprehensive scope of the network meta-analysis, which allowed both direct and indirect comparisons of multiple ICPIs. Previous network meta-analytical attempts have focused on the general immune-mediated adverse effects of ICPIs, potentially diluting the requisite rigor required for reporting the exact burden of this adverse effect. The novelty of our approach is the laser focus on pneumonitis, which, therefore, provides an exact comparative point estimate of its risk in this cohort of patients. The inclusion of monotherapy and combination therapy regimens reflects real-world practice. Robust sensitivity analyses, low heterogeneity, and symmetrical funnel plots further support the credibility of our findings.

However, the aforementioned strength analysis of these data schemes is fraught with lots of limitations. This includes the predominance of early-phase studies and variable pneumonitis definitions. Sparse data for some agents led to wide confidence intervals, and socio-demographic data were often missing or inconsistently reported, limiting detailed subgroup analysis. Additionally, the Common Terminology Criteria for Adverse Events (CTCAE) were used for adverse event grading in nearly all studies. One study used modified criteria, specifically the Immune-related Response Criteria and Common Terminology Criteria (IRE and CTCAE) for Adverse Events, while four studies did not specify the grading approach. This, however, did not significantly impact the final point estimate of the comparative difference on the burden of pneumonitis among ICPI analogs. Additional factors, such as differences in patient population and age disparities, though not explored in our current review, are likely to account for some of the heterogeneity seen on the various point estimates apparent in our meta-analysis.

These factors may account for some of the observed high heterogeneity of our study outcome. Nevertheless, the stability of our point estimates across both direct and indirect comparisons suggests that these limitations are unlikely to significantly impact the burden of pneumonitis vis-à-vis ICPI exposure.

## 5. Conclusions

Our analysis demonstrates that pneumonitis risk varies among ICPIs, with pembrolizumab and sugemalimab showing the highest odds. Although the absolute incidence is low, the potential severity of pneumonitis warrants vigilant monitoring. These results should guide clinicians in risk stratification and treatment planning, and support the development of standardized reporting criteria and further comparative research.

## Figures and Tables

**Table 1:** Socio-demographic characteristics as well as the interventions and proportion of adverse effect in the included studies. The sample sizes across trials varied widely, ranging from as few as 13 participants [21] to over 10,000 [28,41], reflecting both exploratory and confirmatory trial designs. The median age of participants across studies ranged from 50 to 72 years, with most cohorts being predominantly male. Smoking history, although clinically relevant in pneumonitis risk, was inconsistently reported across the included studies.

Study ID	Cancer Type	Phase	Total Sample Size	Age (Median, IQR)	Gender (Male)	ICPI	Follow-Up Duration (Months)	Pneumonitis (Any Grade)—Arm 1 (N)	Pneumonitis (Any Grade)—Arm 2	Pneumonitis (Grade ≥ 3)—Arm 1	Pneumonitis (Grade ≥ 3)—Arm 2	Adverse Event Grading Method (e.g., CTCAE)
Aggarwal 2022 [20]	NSCLC	I/II	133	65 (21–88)	92 (69.2%)	PEM	0.7	8	0	3	0	CTCAE
Altan 2023 [21]	NSCLC	I/II	13	63 (47–81)	5 (38%)	IPI	23	1	0	1	0	CTCAE
Antonia 2016 [32]	SCLC	I/II	216	61 (56–65)	32 (59%)	NIV	9.25	3	2	1	1	CTCAE
Arrieta 2020 [42]	NSCLC	II	78	50.1 (41.2–59.0)	19 (48%)	PEM	8.4	23	5	0	3	none
Armstrong 2024 [43]	NSCLC	II	105	71 (46–79)	9 (69%)	PEM	10.1	1	1	0	1	CTCAE
Bahce 2024 [44]	NSCLC	II	30	64 (43–73)	14 (47%)	IPI	25.8	4	0	2	0	none
Bestvina 2021 [45]	NSCLC	I	37	61.4 (36–78)	21 (56.8%)	NIV	17	0	2	0	2	CTCAE
Chang 2024 [46]	NSCLC	II	156	72 (66–78)	54 (37.8%)	NIV	33	1	2	0	0	CTCAE
Durm 2020 [47]	NSCLC	II	93	66 (45–84)	59 (64%)	PEM	32.2	16	0	6	0	CTCAE
Felip 2019 [48]	NSCLC	II	811	66 (31–86)	640 (78.9%)	NIV	18	38	0	6	0	CTCAE
Fujimoto 2019 [22]	NSCLC	II	18	71.5 (68.5–76.3)	17 (94%)	NIV	14.2	2	0	0	0	CTCAE
Goldberg 2020 [23]	NSCLC	II	42	60 (56–71)	14 (33%)	PEM	8.3	3	0	2	0	CTCAE
Goldberg 2016 [24]	NSCLC	II	18	59 (33–82)	6 (33%)	PEM	6.8	1	0	1	0	CTCAE
Jabbour 2020 [25]	NSCLC	I	21	69.5 (53–85)	10 (48%)	PEM	16	7	0	2	0	CTCAE
Ahn 2022 [26]	NSCLC	Ib	34	57 (44–78)	15 (44.1%)	DUR	20.4	2	0	2	0	CTCAE
Lin 2019 [27]	NSCLC	II	52	67 (50–83)	27 (68%)	ATE	22.5	3	7	0	1	CTCAE
Liu 2023 [28]	NSCLC	III	10,953	66 (59–74)	906 (53%)	ATE	8.3	357	128	54	27	CTCAE
Mattes 2021 [29]	NSCLC	I	35	66 (58.5–70.5)	17 (49%)	ATE	14	9	0	0	0	CTCAE
Peters 2019 [30]	NSCLC	II	80	62 (41–78)	65.(90%)	NIV	13.4	34	0	8	0	none
Rizvi 2015 [31]	NSCLC	II	117	65 (57–71)	85 (73%)	NIV	8	6	0	4	0	CTCAE
Ross 2024 [33]	NSCLC	II	62	63.9 (38.1–86.5)	30 (48.4%)	ATE	31.2	4	0	4	0	CTCAE
Shaverdian 2017 [34]	NSCLC	I	98	65.5 (32.0–83.0)	51 (53%)	PEM	32.5	3	0	0	0	Immune-Related Response Criteria and Common Terminology Criteria for Adverse Events
Welsh 2020 [35]	SCLC	I/II	36	64 (41–79)	16 (40%)	PEM	23.1	6	0	3	0	CTCAE
Wong 2021 [36]	NSCLC	Ib	23	60 (52–67)	14 (60.9%)	IPI	23	6	0	4	0	CTCAE
Chalmers 2019 [37]	NSCLC	I	14	-	-	IPI	3	0	1	0	0	none
Zhou 2022 [38]	NSCLC	III	381	60.5 (55–65)	351 (92%)	SUG	14.3	48	21	8	1	CTCAE
Wang 2022 [39]	SCLC	III	462	62 (56–66.5)	372 (80.5%)	ATE	13.5	4	0	4	0	CTCAE
Malhotra 2024 [40]	SCLC	I/II	36	60 (43–80)	19 (56%)	IPI	2.5	0	0	0	0	CTCAE
O’Brien 2022 [41]	NSCLC	III	1117	65 (58–71)	1041 (88.4%)	PEM	35.6	34	16	7	4	CTCAE

ATE: Atezolizumab; PEM: Pembrolizumab; DUR: Durvalumab; IPI: Ipilimumab; NIV: Nivolumab; SUG: Sugemalimab.

**Table 2:** Showing point estimates of both direct and indirect comparison between various immune checkpoint inhibitors and placebo, as well as indirect comparison between various ICPI analogs.

Comparison	Active	Control	OR [95% CI]
ATE–ADE	ATE	ADE	4.03 [0.45–36.16]
ATE–PLA	ATE	PLA	1.33 [0.38–4.62]
ATE–PEM	ATE	Pembrolizumab	25.45 [1.42–457.06]
DUR–PLA	DUR	PLA	2.06 [0.18–23.88]
IPI–NIV	IPI	NIV	0.15 [0.02–0.97]
IPI–PLA	IPI	PLA	1.93 [0.06–59.87]
IPI–ATE	IPI	ATE	3.71 [0.66–20.76]
NIV–PLA	NIV	PLA	2.69 [0.64–11.35]
PEM–PLA	Pembrolizumab	PLA	2.67 [1.70–4.17]
SUG–PLA	SUG	PLA	2.45 [1.52–3.95]
Indirect ATE vs. Pembrolizumab (2,9)	ATE	Pembrolizumab	0.50 [0.13–1.87]
Indirect DUR vs. Pembrolizumab (4,9)	DUR	Pembrolizumab	0.77 [0.06–9.32]
Indirect IPI vs. Pembrolizumab (6,9)	IPI	Pembrolizumab	0.72 [0.02–23.11]
Indirect IPI vs. Pembrolizumab (7,3)	IPI	Pembrolizumab	94.33 [3.27–2723.93]
Indirect NIV vs. Pembrolizumab (8,9)	NIV	Pembrolizumab	1.01 [0.22–4.55]
Indirect ADE vs. Pembrolizumab (1,3)	ADE	Pembrolizumab	6.32 [0.17–237.83]
Indirect PLA vs. Pembrolizumab (2,3)	PLA	Pembrolizumab	19.20 [0.83–446.11]
Indirect SUG vs. Pembrolizumab (10,9)	SUG	Pembrolizumab	0.92 [0.48–1.77]

Footnote: ATE: Atezolizumab; ADE: Avelumab; PLA: Placebo; PEM: Pembrolizumab; DUR: Durvalumab; IPI: Ipilimumab; NIV: Nivolumab; and SUG: Sugemalimab.

**Scheme A1:**
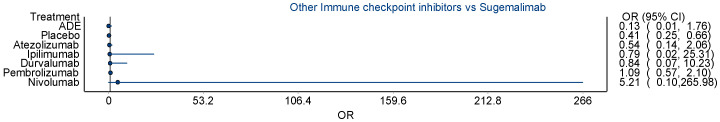
Figure showing comparative estimate between sugemalimab and other ICPIs.

**Scheme A3:**
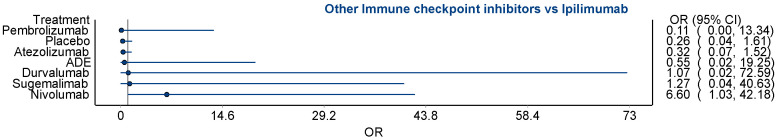
Figure showing comparative estimate between ipilimumab and other ICPIs.

## Data Availability

The original contributions presented in this study are included in the article. Further inquiries can be directed to the corresponding author.
